# The Impact of Robotic Fractionated Radiotherapy for Benign Tumors of Parasellar Region on the Eye Structure and Function

**DOI:** 10.3390/jcm12020404

**Published:** 2023-01-04

**Authors:** Michal Orski, Rafal Tarnawski, Edward Wylęgała, Dorota Tarnawska

**Affiliations:** 1 Department of Ophthalmology, Ludwik Rydygier Memorial Specialized Hospital, Os. Złotej Jesieni 1, 31-826 Cracow, Poland; 2 III Radio and Chemotherapy Clinic, Maria Sklodowska-Curie National Research Institute of Oncology, Branch in Gliwice, Wybrzeże Armii Krajowej 15, 44-100 Gliwice, Poland; 3 Department of Ophthalmology, District Railway Hospital, Panewnicka 65, 40-760 Katowice, Poland; 4 Chair and Clinical Department of Ophthalmology, Faculty of Medical Sciences in Zabrze, Medical University of Silesia, 40-760 Katowice, Poland; 5 Institute of Biomedical Engineering, Faculty of Science and Technology, University of Silesia in Katowice, 75 Pułku Piechoty 1A, 41-500 Chorzow, Poland

**Keywords:** CyberKnife, radiotherapy, radiation, endothelial cell density, RNFL

## Abstract

Purpose: To evaluate the radiation effect of fractionated robotic radiotherapy of benign tumors located in the parasellar region on the anterior and posterior segments of the eye. Methods: A prospective observational study based on the expanded ophthalmological examination. The pre-treatment baseline was used as a control for the post-radiotherapy follow-up examinations. The study group consists of 34 patients (68 eyes) irradiated using the CyberKnife system. There were ten patients with cavernous sinus meningioma, nine with pituitary adenoma, five with meningioma of the anterior and middle cranial fossa, five with meningioma in the region close to optic chiasm, three with craniopharyngioma, and two with meningioma of the orbit. All patients were treated using three fractions of 600–800 cGy. We assessed the impact of radiation on the eye based on changes in anatomical and functional features. The condition of the eye surface, central corneal thickness (CCT), endothelial cell density (ECD), lens densitometry, central macular thickness (CMT), and retinal nerve fiber layer (RNFL) were the anatomical features assessed. The functional tests were best-corrected visual acuity (BCVA), intraocular pressure (IOP), visual field (VF) and visual-evoked potentials (VEP). An ophthalmologic examination was performed before and 6, 12, 18, and 24 months after radiotherapy. Results: We did not observe any significant changes in BCVA, IOP, CCT, CMT, VF, and VEP, nor in the slit-lamp examination during the two-years observation. We found a significant decrease in ECD at all follow-up measurements. The drop in ECD exceeded approximated age-related physiological loss. The reduction in ECD was not large enough to disrupt corneal function and thus affect vision. We also observed a statistically significant reduction of RNFL in all observation time points. However, there was no correlation between the dose delivered to the optic pathway and the decrease in RNFL thickness. The thinning of the RNFL was not significant enough to impair visual function. Conclusion: Fractionated robotic radiotherapy of the tumors located close to the optical pathway is safe and does not impair patient’s vision. Minor changes found in optic nerve anatomy (RNFL thinning) might be related to radiation effect or tumor compression. The causal relation between low doses of radiation delivered to the cornea and the observed significant but slight decrease in ECD is uncertain. The observed changes did not cause visual disturbances perceivable by the patients.

## 1. Introduction

Radiotherapy is particularly useful in the management of tumors of the central nervous system (CNS) that cannot be treated surgically and of lesions located in the unfavorable areas where the risk of surgical intervention overwhelms the benefits for the patient. A substantial amount of benign CNS tumors progress slowly, showing only a few symptoms, and do not affect the patient’s quality of life significantly. These patients require minimally invasive and advanced methods of treatment. The location of benign lesions in the proximity of the eye and the risk of radiation-associated complication make it difficult to choose and commence a proper treatment. Radiation-induced ocular complications such as cataract, dry eye syndrome, corneal erosions, perforations, and scarring have been reported [1,2]. Serious and potentially irreversible complications such as radiation retinopathy and neuropathy or neovascular glaucoma may occur several years after exposure to the radiation [2,3]. CyberKnife (CK) radiotherapy, introduced in 1994, is one of the most advanced treatment modalities. High precision and simultaneous imaging enable a substantial reduction of the radiation delivered to healthy tissues. CK radiotherapy is a relatively new therapy, and there are very few reports regarding its impact on the eye. The biomedical effects of hypofractionated radiotherapy differ from the effects of conventional radiotherapy and radiation-induced side effects on healthy tissues beyond the targeted area need to be studied carefully. Therefore, in this study, we aimed to evaluate the influence of the CK radiotherapy on the anterior and posterior segments of the eye. To our knowledge we present the first prospective study using the most advanced ophthalmology techniques to explain the effects of hypofractionated radiotherapy on the optic apparatus structure and function. The most relevant part of study applies to optic nerve and chiasm, as the highest radiation doses were used, but we also studied the impact of low doses on the structure and function of the anterior segment of the eye.

## 2. Materials and Methods

### 2.1. Participants

We prospectively observed patients undergoing CK radiotherapy in the 3rd Radio and Chemotherapy Clinic for a period of 24 months. Thirty-four patients, for whom CK radiotherapy was planned in the Department of Radiotherapy Maria Sklodowska-Curie National Research Institute branch in Gliwice, were enrolled in the study. There were 26 women (76.5%) and 8 men (23.5%). The male:female ratio for meningioma patients in MSC Institute is 3:1 (calculated from 400 consecutive patients). The study group included 10 patients with cavernous sinus meningioma, 9 with pituitary adenoma, 5 with meningioma of the anterior and middle cranial fossa, 5 with meningioma in the region close to optic chiasm, 3 with craniopharyngioma, and 2 with meningioma of the orbit. Before radiotherapy and at designated intervals after its completion, patients had an enhanced ophthalmological examination performed at the Department of Ophthalmology of District Railway Hospital in Katowice. Follow-up visits were scheduled at 6, 12, 18 and 24 months after radiotherapy. Three patients missed an ophthalmological examination at six months after irradiation but attended the next follow-up exams, 31 patients had examinations after 12 months, 27 patients had examinations after 18 months and 25 patients completed a 24-month ophthalmological follow-up. None of the patients had undergone ocular surgery or had known ocular disease at the time of enrollment. Also, during the two-year follow-up period, none of the patients underwent eye surgery.

### 2.2. Radiation Therapy

**Figure 1:**
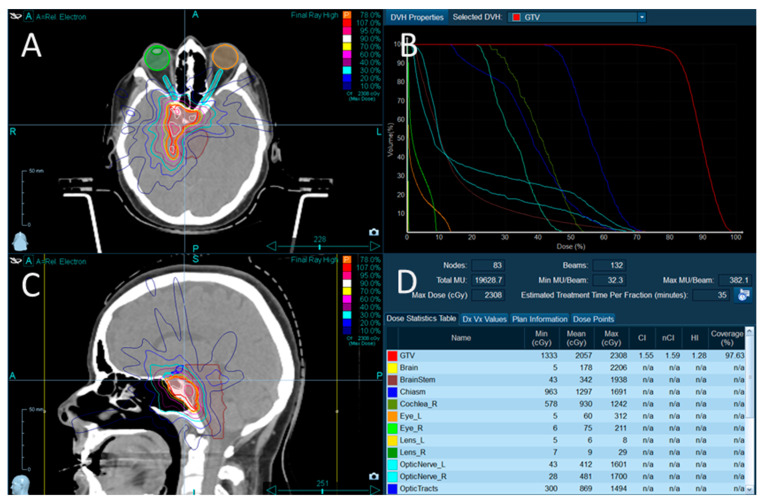
Example picture of irradiated target-meningioma. (**A**). Illustration of isodoses, contours of tumor, optic pathway, the brain stem. Axial view. (**B**). Dose-volume histogram. Illustration of doses given to different volumes of tumor and organs at risk. (**C**). Sagittal view of isodose distribution. (**D**). Minimal, mean, and maximal doses delivered to tumor and organs at risk.

Radiation doses were estimated using the CK Treatment Planning System. All structures were contoured using the magnetic resonance imaging technique in combination with the computed tomography imaging for dose calculations (Figure 1).

MRI/CT image registration was done using a rigid algorithm included in the Cyber Knife treatment planning software. Rigid registration is usually best for cranial targets as we do not expect anatomy changes between CT and MRI imaging. Doses calculated for optic nerves, chiasm, and lenses are most accurate as these structures are visible on MRI/CT imaging and are included in dose optimization. Doses calculated to lenses were used as a surrogate for cornea doses. Precise contouring of the cornea is impossible as it is too thin. Additionally, the cornea is situated on the eye’s surface, so the high energy electrons do not reach equilibrium in this organ, adding to the dose calculation uncertainty. Dose to lens is a better estimate of a dose to cornea than the dose calculated to cornea by the treatment planning system. The maximal dose calculated to the eye globe was used as the best surrogate for retinal dose estimation. We made this assumption because of the optimization algorithm included in the CyberKnife software (Multiplan 4.6 Accuray, Sunnyvale, CA, USA). Reducing the dose to the eye, controlled by the attached algorithm, causes the retina to receive the highest dose among all eye structures because it is at the location closest to the irradiated area. Lacrimal glands were not included in the optimization of treatment plans because of the central position of the irradiated tumors. We do not present the exact doses for the lacrimal glands.

Doses of radiation calculated for the eye structures and the optic pathway are presented in Table 1. All treatment plans were designed to fulfill dose-volume criteria for the optic pathway. All patients were treated using three fractions of 600–800 cGy.

Data on 68 eyes (34 patients) were analyzed. The results obtained during follow-up visits referred to the results of the same eyes collected before radiotherapy, which were treated as a control group.

### 2.3. Ophthalmologic Examination

The radiation-induced impact on the anterior and posterior segments of the eye was evaluated based on the changes in several anatomical and functional features.

In addition to the slit lamp examination, the following quantitative measurements were performed at each follow-up visit: central corneal thickness (CCT), endothelial cell density (ECD), lens densitometry, central macular thickness (CMT), and retinal nerve fiber layer thickness (RNFLT). The CCT measurement was obtained with optical coherence tomography (OCT Visante, Carl Zeiss Meditec Inc., Dublin, CA, USA). The ECD was calculated using a non-contact specular microscope (Topcon SP-3000P). The endothelial cells morphology was analyzed in the scans obtained in every follow-up visit, and a 50% cut-off value was used in the assessment of the prevalence of hexagonal cells. The Pentacam Nucleus Staging (PNS) software of the OCULUS Pentacam^®^ device was used to objectively measure the density of the lens and assess the advancement of the cataract. CMT and RNFL were measured with optical coherence tomography (Cirrus SD-OCT, Carl Zeiss Meditec, Inc., Dublin, CA, USA).

To assess the functional features of the eye, the best-corrected visual acuity (BCVA), intraocular pressure (IOP), visual field (VF), and visual evoked potentials (VEP) were analyzed. BCVA was examined using Snellen charts and the results were subsequently converted to logMAR equivalent. IOP was measured with Goldmann’s applanation tonometry. The VEP parameters analyzed were P100 wave latency and amplitude. VEP were measured with the Metrovision MonElec2 system (Metrovision, Perenchies, France). The atient visual field (VF) was evaluated by standard automated perimetry using static automated white-on-white threshold perimetry (SITA Standard 30−2, Humphrey Field Analyzer II; Carl Zeiss Meditec Inc., Dublin, CA, USA). A visual field was defined as reliable when fixation losses and false-positive and false-negative errors were less than 20%. Average visual field sensitivity was expressed in Mean Deviation (MD) and Pattern Standard Deviation (PSD) results, as calculated by the perimetry software.

### 2.4. Statistical Analysis

The pre-treatment baseline was used as a control for the post-radiotherapy follow-up examinations. Statistical significance was calculated from the differences between the values before and after treatment, not from differences between means. Statistical significance was calculated using the student’s *t*-test for dependent groups with STATISTICA 13.3 software.

## 3. Results

In the 24-month observation, there were no statistically significant changes in the BCVA (mean logMAR 0.15 SD = 0.41). Only one patient worsened from logMAR 2 to 3, and for this patient dose to chiasm and optic nerves was lower than 9 Gy in three fractions, which is a low dose.

No changes of the anterior and posterior segments of the eye were observed in the slit-lamp examination. We also did not find a significant difference in CCT and lens density. We observed a significant decrease in ECD for all observation timepoints when compared to ECD before radiotherapy (Table 2), but we did not observe a correlation between ECD loss and dose delivered to the cornea. A 50% cut-off value was used to assess the prevalence of hexagonal cells in the corneal endothelium and was found to be greater in all examination scans.

**Figure 2:**
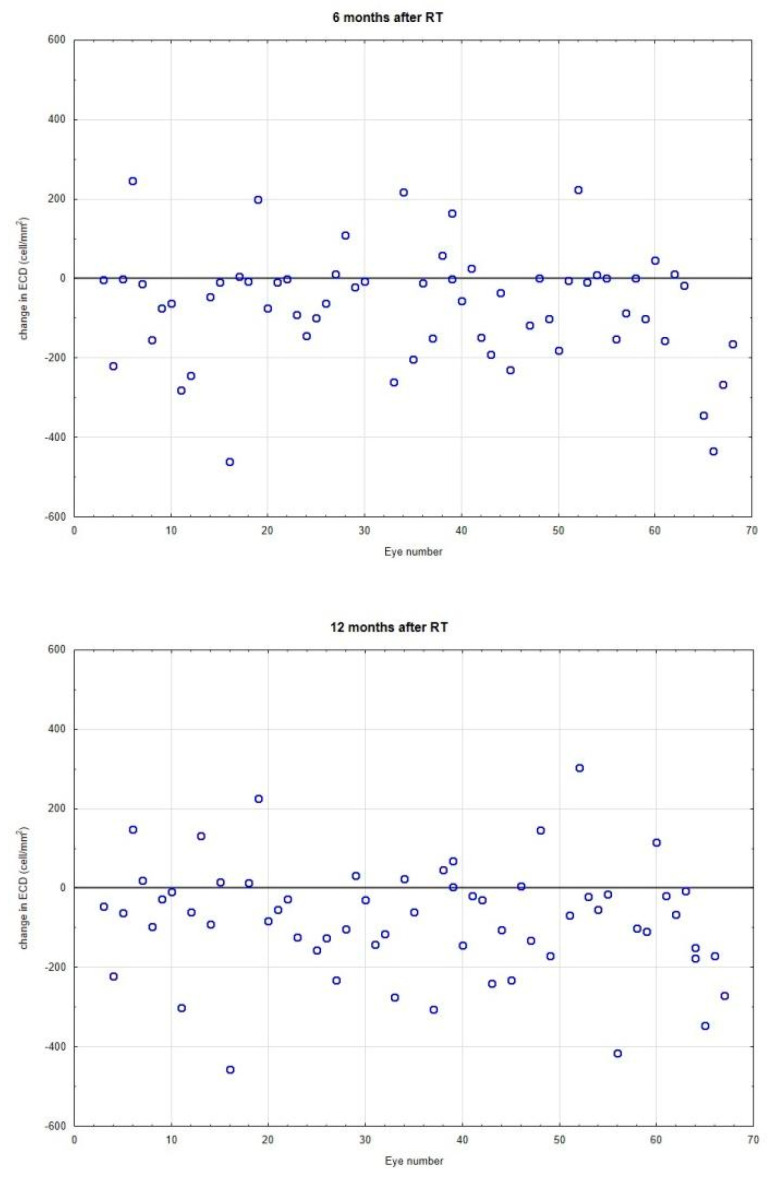

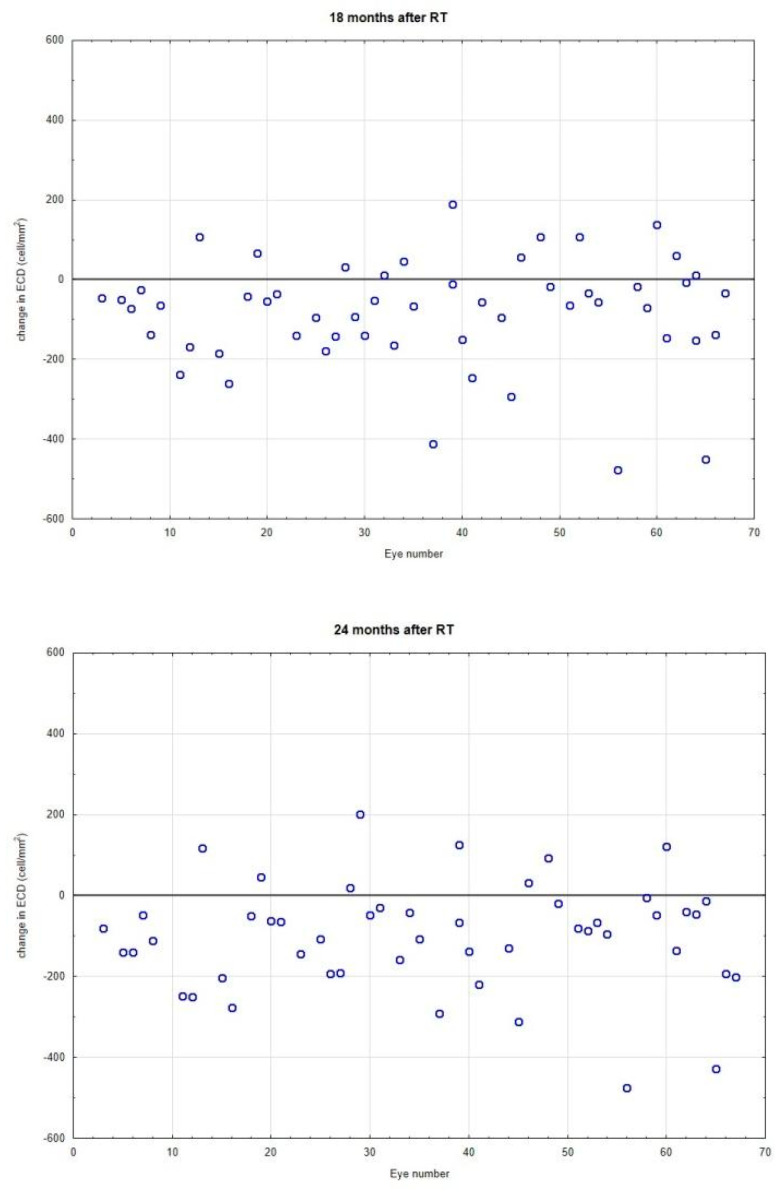
Changes in endothelial cell density (ECD, cells/mm^2^) 6, 12, 18 and 24 months after irradiation. Each point represents a difference in ECD measurement value for the individual eye. Line 0 represents the same values. Almost all patients had a reduction of ECD.

We observed a change in endothelial cell density for all observation time points compared to pre-irradiation values, as illustrated in Figure 2. In addition, a significant decrease in ECD was also noted between 6 and 24 months of observation.

There were no significant changes in the IOP, visual field MD and PPSD values. With the posterior segment OCT imaging we did not observe significant change in CMT measurements.

We observed a statistically significant thinning of RNFL at all observation timepoints (Table 3).

There were six eyes in five patients for whom the decrease of RNFL thickness was more than 10%. We did not observe a significant correlation between dose to optic pathway and decrease of RNFL. Doses delivered to individual parts of optic pathways were not significantly different for eyes with a drop of more than 10% of RNFL, when compared to other eyes.

**Figure 3:**
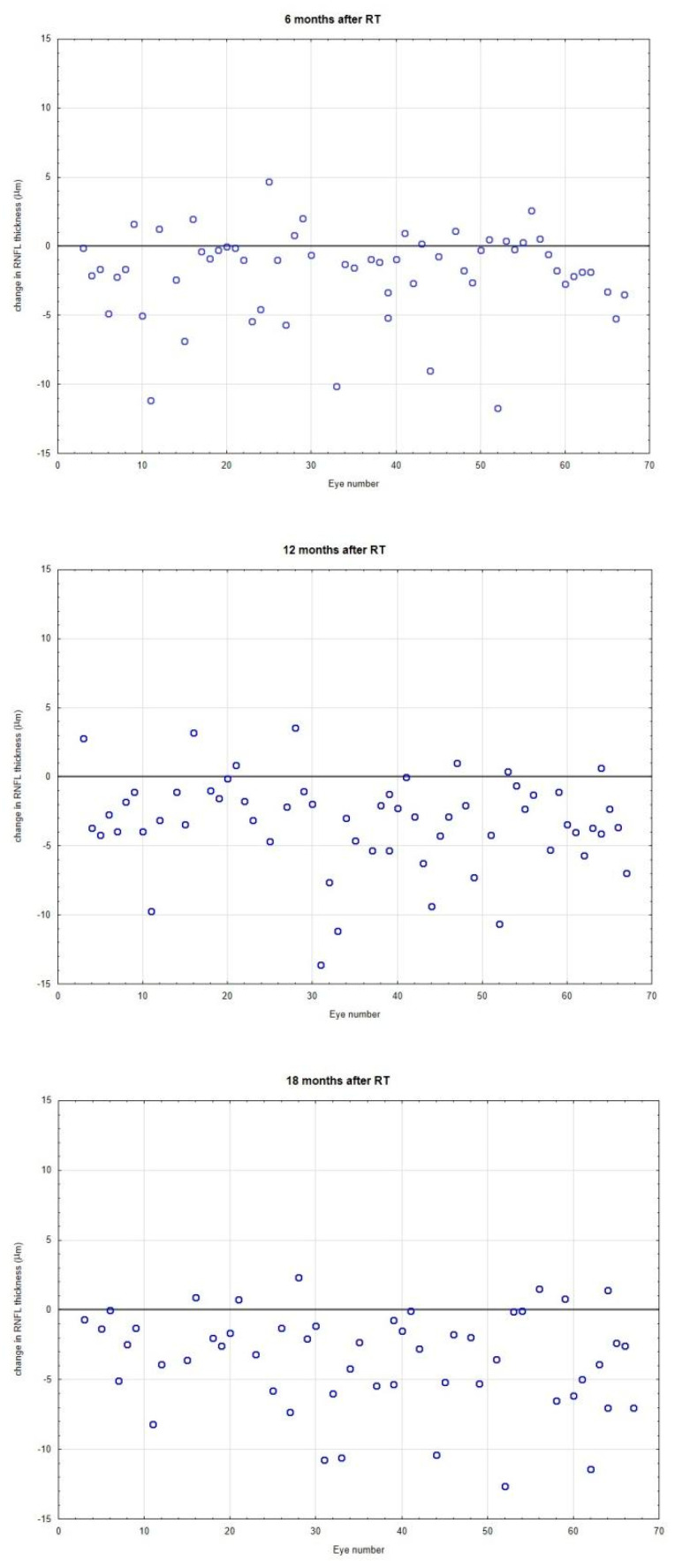

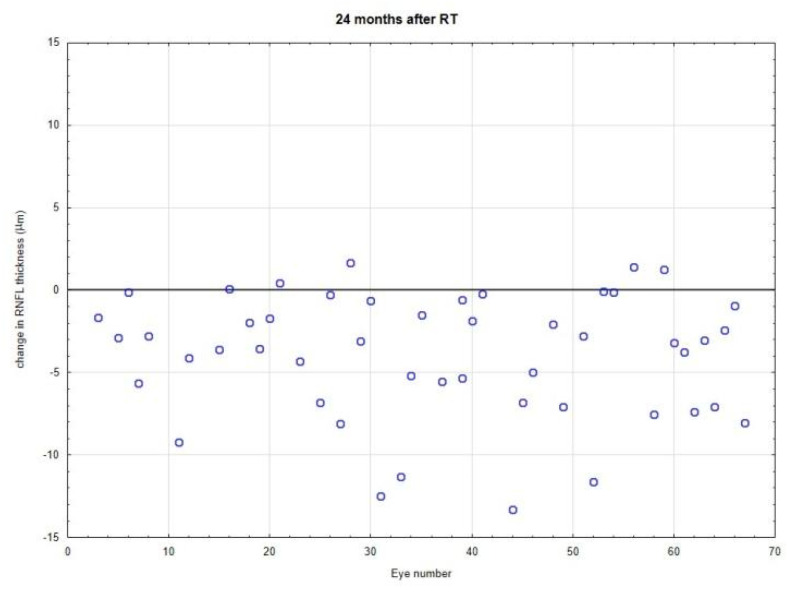
Changes in retinal nerve fiber layer (RNFL, µm) thickness at 6, 12, 18, and 24 months after irradiation. Each point represents a difference in RNFL measurement value for the individual eye. Line 0 represents the same values. Almost all patients had a reduction of RNFL.

We observed ECD changes at 6, 12, 18 and 24 months after irradiation, as illustrated in Figure 3.

## 4. Discussion

Radiation-induced complications remain the most important limiting factor for commencing radiotherapy. Hypofractionated radiotherapy using high doses is relatively new. We decided to evaluate the group of patients treated with CK radiotherapy using advanced ophthalmological examination. We selected for our study a group of patients qualified for CK radiotherapy because of benign tumors located in close vicinity of the optic pathway. We could have expected radiation damage to the optic nerve, chiasm, or lens. Radiation doses delivered to the retina and cornea are relatively low, so we did not expect radiation damage to these structures [4]. The major strength of our study is the ophthalmological examination undertaken before the radiotherapy. By performing ophthalmological tests prior to irradiation, we were able to establish patient-specific optical performance baselines, thereby gaining a more precise understanding of the individual patient’s net outcome. Statistical significance was calculated between the values established before treatment and values obtained during the period of two-year observation.

The majority of corneal erosions are caused not by the radiation damage to epithelium but by the dry eye syndrome caused by radiation damage to the lacrimal gland [1]. We do not expect this effect for CK radiotherapy because lacrimal glands are not irradiated in substantial doses. Data for corneal tolerance varies significantly, but most publications claim that doses in the range of 30–50 Gy lead to corneal erosions, 40–50 Gy cause corneal edema, and doses exceeding 65 Gy lead to corneal perforations [1,3]. For our patients doses to cornea were estimated to be equal to doses calculated for the lens. A mean dose of 14 cGy and maximal dose of 70 cGy are extremely low, and we did not expect any changes to cornea. In this study, we did not observe any radiation related changes on the surface of the eye during the biomicroscopic examination.

In addition to the eye surface evaluation, the impact of the irradiation on the cornea was assessed based on the ECD, the prevalence of the hexagonal cells in the corneal endothelium, and CCT. We found statistically significant ECD loss at all follow-up timepoints compared to pre-irradiation values. The decrease in ECD was 1.9% SD 0.5%. A significant decrease in ECD was also observed between six and 24 months of follow-up. There was no significant correlation between the radiation dose delivered to the cornea and the change in ECD at any timepoint. Since the study lasted for two years, the physiological loss of ECD that could have occurred at that time was considered. The observation that ECD decreases with age has been confirmed in many studies and physiological endothelial cell loss is estimated at a rate of approximately 0.3–1.0% per year [5,6,7,8,9]. It has been reported to be 0.3% and 0.5% per year in the population of Denmark [7] and New Zealand [8], respectively. Cheng reported it to be 1.0% [9]. Thus, even considering the physiological decline in ECD, the decrease observed in the study was significantly greater than that resulting from aging alone.

ECD and cell morphology are key factors affecting decision-making and influencing short- and long-term results in every surgery related to the anterior segment. Surgically induced loss of ECD is estimated to be in a range of 0–30% for any anterior segment surgery [10,11,12,13,14]. A minimum ECD of 600–1200 cells/mm^2^ and more than 50% prevalence of hexagonal cells are the widely accepted criteria required for a safe anterior segment surgical procedure; however, more strict criteria may apply for phakic intraocular lens implantations [10,12]. Suranyi et al. reported the possible impact of radiation on corneal endothelium [15]. Significant loss of ECD was observed in patients undergoing brachytherapy for choroidal melanoma, in which ECD decreased from 2147 ± 128 cells/mm^2^ to 2050 ± 108 cells/mm^2^ in six months [15]. However, Razzaq et al. did not show any immediate change in ECD in patients treated with brachytherapy for choroidal melanoma but reported an increased loss of ECD in those patients who subsequently underwent uneventful cataract surgery [16]. Radiation doses delivered to the cornea during brachytherapy were in the range of 234–675 Gy [16]. However, in the Razzaq study, radiation doses delivered to the cornea were over one thousand times greater than those used in our study, as the latter were equal to approximately 15.76 cGy. Unfortunately, there are no studies assessing the effect of lower doses of radiation on the endothelium than those used during brachytherapy for choroidal melanoma. The prevalence of hexagonal cells in the corneal endothelium in our study was found to be greater than the 50% cut-off value in all of the examinations performed. We did not observe any change in CCT during the 24-month follow-up.

The practical importance of observed reduction of the ECD is unknown. Considering its extent seems unlikely to affect the course of anterior segment surgery. Radiation doses related to the observed changes are small and generally are thought to be safe for proliferative tissues such as the mucosa. The effect of radiation may be more visible in tissues where proliferation is slow or, as in the case of the endothelium, maintained in the non-proliferative state. Regarding this, a further assessment of the effect of ionizing radiation on ECD is required.

The dose tolerated by the lens was previously reported to be in the range of 8–10 Gy in the 1990s and gradually lowered to 2 Gy until 2011 [17]. More recent publications indicate that doses as low as 500 mGy can lead to lens opacification and cataract formation [18]. The mean dose calculated for lenses delivered for our patient group was 14 cGy and the maximal calculated dose was 70 cGy. The human lens is one of the most radiation-sensitive tissues and a few recent studies have shown that the tolerated doses might be even lower than what has been described in previous studies [16,18,19,20,21,22,23,24]. The International Council on Radiation Protection Guidelines and the tolerated dose has been recently revised to 0.5 Gy/year for professionals who expose themselves to radiation [17]. The incidence of cataract formation is estimated to be 33% in eight years in individuals receiving doses in the range of 2.5–6.5 Gy and 66% in four years in those receiving 6.5–11 Gy [1]. Cataract formation was observed six months after exposure to radiation at a dose of 5 Gy [1]. In this study, the status of the crystalline lens was assessed based on the bio-microscopic examination, best corrected visual acuity (BCVA) measurements, and most importantly by lens densitometry. We did not observe any detectable changes in the status of the lens using slit-lamp biomicroscopy. There was no statistically significant difference in the BCVA values at any follow-up visit (*p* > 0.05). Pentacam Nucleus Staging (PNS) software was used to objectively quantify lens density. In previous studies, lens status was measured based on subjective judgment or with the use of lens opacification grading systems, for example, the Wisconsin system, the Wilmer system, the Oxford system, and the Lens Opacities Classification System III (LOCS III), with the latter being most widely accepted. The PNS system is the first system providing clinicians with detailed lens densitometric measurements based on the mathematical model, which enables precise follow-up with subsequent data. Based on the lens densitometry PNS grades, the lens status score ranges from 0 to 5. More advanced grading means a denser and less transparent crystalline lens. In this study, there were no statistically significant changes in the lens status. We showed that D_max_ of 70 cGy did not cause cataract formation or progression.

Radiation retinopathy is a serious complication that presents typically six months to three years following treatment, however delayed manifestations may occur even after 15 years. The threshold dose is estimated to be 30 Gy with the incidence increasing dramatically with doses over 50 Gy [1]. With total doses over 60 Gy, the estimated risk of developing radiation retinopathy is 50% and rises up to 85–90% with total doses in the range of 70–80 Gy [25]. Total doses below 35–60 Gy are considered safe; however, the incidence of radiation retinopathy was described with doses as low as 11–17 Gy [25,26,27,28,29,30,31,32,33]. In our group the mean maximal dose was 130 cGy with the maximal dose 750 cGy delivered to peripheral parts of the eye. This is at the dose range below the threshold of retinal damage. CMT is a sensitive parameter in the assessment of retinal anatomy in the macular region and can detect macular changes at a subclinical stage. Both increased and decreased CMT values may indicate macular involvement. Increased values of CMT may manifest increased vascular permeability leading to macular edema, whereas decreased CMT values may indicate hypoperfusion damage. The mean values of CMT for studied eyes was 168 (SD = 20) and have not changed during the observation period. Stable BCVA, no fundus changes, and stable CMT values indicate no evidence of radiation induced damage to the retina in the 24-month period.

Radiation-induced optic nerve neuropathy (RION) is a devastating and potentially blinding condition which may occur months to years following treatment. Roden and his group recommend 45 Gy as the maximal acceptable total dose [34]. The risk of RION increases with total doses over 55 Gy; however, the incidence of RION was reported with doses not exceeding 11 Gy [1,3]. Single doses lower than 8–10 Gy are considered safe in patients undergoing single and multi-fraction stereotactic radiotherapy. [4,35,36,37]. The dose per fraction received seems to be the most important factor and should not exceed 1,9 Gy [1]. In our study the mean maximal doses were: 1220 cGy SD = 510 cGy for the chiasm and 930 cGy SD = 580 cGy for optic nerves. The maximal calculated doses were 2224 cGy for optic nerves and 2227 cGy for chiasm. Visual evoked potentials analysis, visual field testing, and retinal nerve fiber layer were used to evaluate the potential impact of CyberKnife radiotherapy on the optic tract. VEP was employed to possibly detect subclinical functional changes, whereas RNFL assessment was used to detect subtle anatomical changes. Retinal nerve fiber layer thickness change may precede a visual field defect. OCT was used to measure each patient’s RNFL every six months. We observed a statistically significant decrease of RNFL in all observation timepoints. There were six eyes in five patients for whom the decrease of RNFL was greater than 10%. We did not observe significant correlation between dose to optic pathway and decrease of RNFL. Doses delivered to parts of the optic pathways were not significantly different for eyes with a drop of RNFL of more than 10% compared to other eyes. The observed changes in RNFL may be caused not only by the influence of radiation. In most of the examined patients, the tumor was located in the immediate vicinity of the optic nerve, and these changes could also be caused by tumor compression.

Visual field analysis did not show any change, and optic disc assessment was unremarkable within 24 months. We regard VEP P wave amplitude and latency as a sensitive marker for the integrity and conductivity of the optic nerve. Leber and his group showed that VEP may show abnormalities in anterior visual pathway such as decreased wave amplitude and increased latency long before any symptoms occur [37]. P100 wave amplitude and latency for two different stimuli (7′ and 30′) was analyzed in our study. There were no statistically significant changes in P100 wave latency (*p* > 0.05) and amplitude (*p* > 0.05) for two different stimuli (7′ and 30′). Concerning unchanged BCVA, VFs, the appearance of the optic nerve head, and the only statistically significant decrease in the RNFL value, it can be assumed that the possible damage to the optic nerve found in our study was at a minimal, borderline detectable level.

In conclusion, CK is a very precise and advanced treatment modality that is widely used due to its safety profile. We analyzed the impact of doses delivered to the eye along with the visual pathway in patients treated with CK for CNS tumors. During the 24-month observation, we did not find any statistically significant changes in the BCVA, IOP, CCT, lens densitometry, CMT, VF and VEP. No changes were observed in the slit-lamp examination. In the anterior segment of the eye, we found only statistically significant ECD loss at all observational timepoints, but there was no correlation between the radiation dose delivered to the cornea and the change in ECD. Few previous studies evaluated ECD after radiotherapy, so this assessment was all the more needed. Concerning unchanged BCVA, VFs, the appearance of the optic nerve head, and only a statistically significant decrease in the RNFL value, we can assume that the possible damage to the optic nerve found in our study was at a minimal, borderline detectable level. The radiation dose delivered to individual structures of the eye and optic pathway during CK radiotherapy is low. Still, the biological effect of hypofractionated radiotherapy differs from conventional treatment and therefore should be analyzed carefully. We believe that our findings may help in the proper treatment planning and changing treatment algorithms, as there are technical possibilities to reduce the doses delivered by CK to the eye without compromising the dose delivered to the tumor if it is proven necessary.

## Figures and Tables

**Table 1:** Doses delivered to various parts of the eye were not homogenous. The table shows statistics from maximal doses delivered to specific parts of the eye and optic pathway.

Maximal Doses to Eye Structures	N	Mean (cGy)	SD	Median (cGy)	Maximum (cGy)
right lens & right cornea surrogate	34	19.6	15.3	17	70
right eye & right retina surrogate	34	139.1	135.8	103	757
right optic nerve	34	979.0	623.1	904	2195
left lens & left cornea surrogate	34	17.4	12.9	18.5	71
left eye & left retina surrogate	34	117.9	79.2	104.5	363
left optic nerve	34	884.7	536.6	685.5	2227
chiasma	34	1220.8	513.3	1386	2224

**Table 2:** Changes in endothelial cell density (ECD, cells/mm^2^) between baseline and measurements at 6, 12, 18 and 24 months after irradiation.

	Mean (Cell/mm^2^)	SD	N	Difference (95% Conf. Interv.)	*p* Value
ECD 0	2560	259.9			
ECD 6 mos	2492	267.8	62	67.5 (31.5–103.4)	0.000388
ECD 0	2589	256.2			
ECD 12 mos	2496	247.5	62	93.0 (56.3–129.7)	0.000004
ECD 0	2582	266.5			
ECD 18 mos	2482	244.0	54	100.5 (61–140.1)	0.000005
ECD 0	2580	264.8			
ECD 24 mos	2460	234.0	50	119.9 (79.9–159.9)	1 × 10^−6^

**Table 3:** Changes in retinal nerve fiber layer (RNFL,µm) thickness between baseline and measurements at 6, 12, 18 and 24 months after irradiation.

	Mean (μm)	SD	N	Difference (95% Conf. Interv.)	*p* Value
RFNL 0	90.2	18.8			
RFNL 6 mos	88.2	18.8	60	1.9 (1.1–2.7)	0.000018
RFNL 0	90.8	18.2			
RFNL 12 mos	87.6	18.1	60	3.2 (2.4–4.1)	1 × 10^−8^
RFNL 0	91.8	18.3			
RFNL 18 mos	88.2	18.1	53	3.5 (2.6–4.5)	1 × 10^−7^
RFNL 0	91.9	18.1			
RFNL 24 mos	88.0	18.0	49	3.9 (2.8–4.9)	1 × 10^−7^

## Data Availability

The data are not publicly available due to privacy and ethical considerations.
